# Polymers for the treatment of Alzheimer’s disease

**DOI:** 10.3389/fphar.2025.1512941

**Published:** 2025-01-29

**Authors:** Yunfeng Zhu, Haibin Xu, Chaoyan Yu, Wenting Jiang, Xiaowen Hou, Mingyue Ma, Ji Wu

**Affiliations:** ^1^ The Second Clinical Medical College, Shenyang Medical College, Shenyang, China; ^2^ Department of Neurology, Juntendo University, Tokyo, Japan; ^3^ The First Clinical Medical College, Shenyang Medical College, Shenyang, China; ^4^ School of Public Health, Shenyang Medical College, Shenyang, China

**Keywords:** Alzheimer’s disease, polymer, drug delivery systems, psychiatry, nanoparticle

## Abstract

Alzheimer’s disease (AD) is one of the most common diseases of the central nervous system in the middle-aged and elderly population. It is a neurodegenerative disorder, and its main clinical symptoms include the loss of established memories, a decline in learning capacity, and the buildup of β-amyloid peptides. The disease is often accompanied by neurodegenerative changes and the formation of neurofibrillary tangles. However, the number of drugs available for the clinical treatment of AD remains limited. Currently, existing medications are not effective in completely curing the disease or stopping its progression. Due to their excellent biocompatibility and biodegradability, polymers have been widely used as drug delivery carriers in various fields including cancer therapy and wound healing. The use of polymers enables targeted drug delivery and prolonged release profiles. In recent years, researchers have made significant progress in utilizing polymers such as polyethylene glycol, poly (lactic-co-glycolic acid) (PLGA), and chitosan (CS) to deliver drugs and blood-brain barrier receptor ligands for the treatment of AD. Moreover, many polymers with inherent therapeutic properties have been developed, including the already marketed GV-971 as well as experimental polymers such as PLGA and CS oligosaccharide. This review summarizes the applications of polymers in AD treatment over the past few years and highlights their current limitations to help researchers better understand current advancements in polymer development and identify future research directions.

## 1 Introduction

According to epidemiological surveys, there are more than 50 million patients with Alzheimer’s disease (AD) worldwide, and this number will triple by 2050 (Scheltens et al., 2021). AD is by far the main cause of dementia in people over the age of 60 years. In the first few decades after a Bavarian psychiatrist Alzheimer discovered AD, researchers made little progress in understanding the pathological changes of this disease. In the 1960s, with the advent of electronic microscopy, researchers were able to observe senile plaques and neurofibrillary tangles. Subsequently, additional pathological changes associated with AD were documented, analogous to the appearance of mushrooms following a precipitation event (Aston-Jones et al., 1985; Xu et al., 2020; Greenamyre and Young, 1989; Saito et al., 1993). To date, a growing number of people regard AD as a syndrome caused by a collection of neuropathological changes rather than a simple disease. Against the backdrop of this setting, Jack and colleagues proposed a new bio-diagnostic hallmark of AD neuropathology, namely, beta-amyloid deposition, phosphorylated tau and neurodegeneration (Jack et al., 2018). The three pathological changes are referred to as AT(N). The diagnosis of AD requires the simultaneous presence of Aβ plaques and tau protein aggregation. Neurodegeneration is often found, but is not necessary for the development of AD. In addition to these three most typical changes, there are also mitochondrial redox abnormalities, N-methyl-D-aspartic acid receptor (NMDAR) position shift, and acetylcholine transmitter release obstacles (Kerr et al., 2017; Babaei, 2021; Saxena and Dubey, 2019). The therapeutic effects of several drugs that have been put into clinical practice are limited, and they cause various peripheral adverse reactions due to the lack of effective brain targeting methods (Birks and Harvey, 2018; Birks and Grimley Evans, 2015; Reisberg et al., 2003; Loy and Schneider, 2006).

**FIGURE 1:**
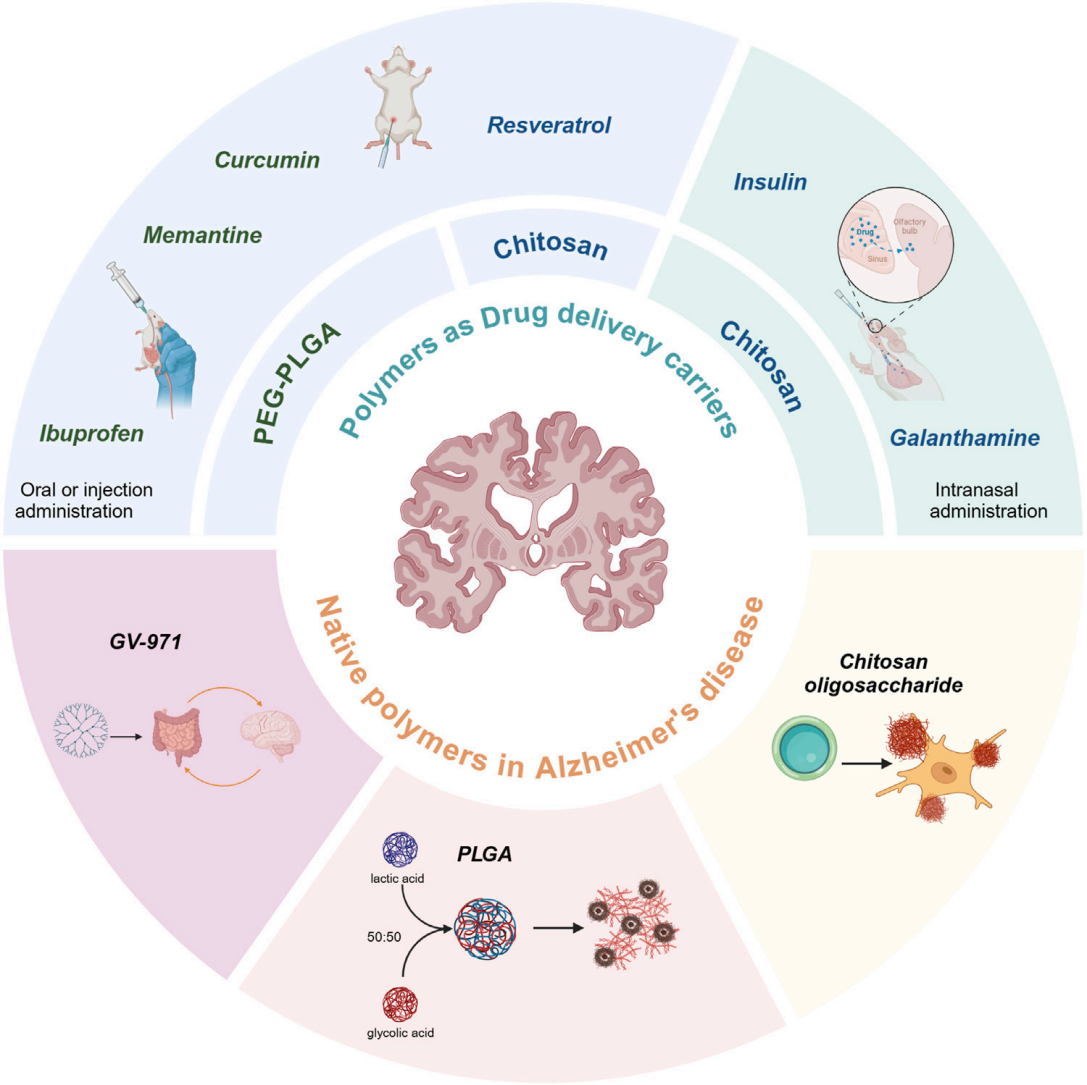
The types of drug-loaded polymers for the treatment of Alzheimer’s disease.

Nanoparticles (NPs) made of polymers are a type of particles with a particle size between 10 and 1,000 nm, which can be loaded with active compounds for drug delivery by adsorption or intranuclear encapsulation (Hettiarachchi et al., 2019). Nanospheres are based on a continuous polymeric network in which the drug can be retained inside or adsorbed onto their surface. The smaller particle size and better sealing of NPs can help drugs cross the blood-brain barrier (BBB) in the body or extend the sustained release curve and reduce the effect of drugs in tissues outside the central nervous system (CNS). In particular, block copolymers have both hydrophilic and hydrophobic activities and offer significant advantages in drug encapsulation efficiency (Ekladious et al., 2019; Cabral et al., 2018). These can bind to resveratrol (Res), curcumin (Cur), insulin, ibuprofen, and other medications. The high hydrophilicity, and soft consistency prevent them from being coupled to macromolecules or degraded by enzymes to improve the bioavailability of the drug and reduce the metabolism in the liver. The advantages of some natural polymers such as low cost and few peripheral side effects have been developing rapidly in recent years, and since its introduction in 2019, GV-971 has been rapidly becoming more widely available on the basis of these advantages (Wang et al., 2024). This review summarizes the progress of AD related polymer drug delivery, as well as the research findings on polymers as drug therapy. The types of drug-loaded polymers are shown in Figure 1.

## 2 Pathophysiology of AD

**FIGURE 2:**
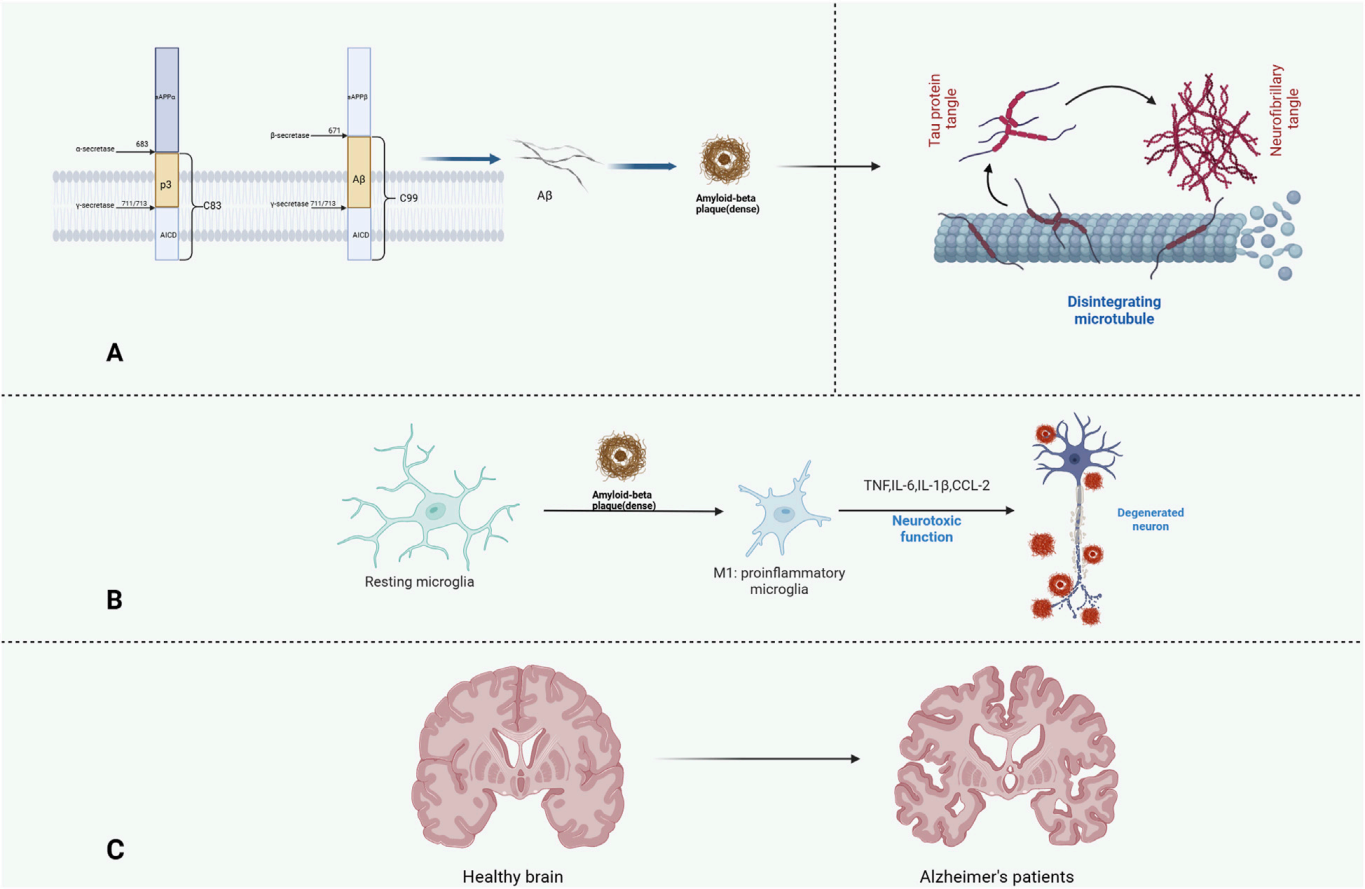
Amyloid plaques, tau phosphorylation and neuroinflammation in Alzheimer’s disease. **(A)** Amyloid precursor protein differently shears to produce Aβ and further deposits to form Aβ plaques. **(B)** Neuroinflammation and neurodegeneration due to cytokine release from microglia activation induced by Aβ plaques. **(C)** Brain shrinkage in patients with Alzheimer’s disease.

The pathogenesis of AD has not yet been fully explored, but Aβ is considered to be an important factor. This substance is derived from the amyloid precursor protein (APP), a neuronal membrane protein. Abnormal postsynaptic acetylcholine receptor locations and excessive sprouting of nerve endings have been reported in APP-deficient mice. These clues suggest a role for APP in neural development (Wang et al., 2005). APP is degraded during metabolism by either α-secretase and γ-secretase or β-secretase and γ-secretase, the latter produces two different amino acid chain lengths, namely, Aβ_40_ and Aβ_42_, depending on the shear position of γ-secretase (Huse et al., 2002). Aβ_42_ is thought to be the more neurotoxic sequence among the two. The detailed shearing process is shown in Figure 2. In summary, Aβ_42_ accumulates in the brain, forming amyloid plaques. This is followed by the activation of glial cells and the pathological phosphorylation of tau (Andronie-Cioara et al., 2023). During this process, Aβ is recognized by pattern recognition receptors in microglia, which produce neurotoxic cytokines and chemokines, such as CCL-4, TNF, IL-6 and IL-1β. This illustrates the importance of neuroinflammation in the development of AD pathology (Martin et al., 2017). Furthermore, Aβ has been demonstrated to impede long-term potentiation (LTP) and dendritic spine density in a manner that is dependent on the activation of NMDAR. The aberrant activation of extrasynaptic NMDARs has been identified as a significant contributor to the substantial decline in synapse number observed in patients with AD (Fani et al., 2021).

## 3 Characteristics of polymer carriers

Polymers are macromolecular compounds formed by the linking of a large number of repeating units through chemical bonds. These assemblies are then subjected to further processing to yield nanomicelles, vesicles, polymers and other products (Feng et al., 2017). Polymers can have one or more of these forms at the same time. Structurally, they can be composed of a single monomer arranged in a repeating manner, or a variety of monomers of different structures arranged in a random alternating manner, with a high degree of customization and desirable physicochemical properties such as solubility, amphiphilicity, and biodegradability (Agrahari and Agrahari, 2018).

Since Abuchowski et al. first coupled monomethoxy-polyethylene glycol (mPEG) to bovine serum albumin in 1977, researchers have found that it is possible to couple polymers to organic or inorganic drugs. This approach was first approved by the US FDA in 1990 (Moncalvo et al., 2020). Although only PEG couplers are commercially available today, much progress has been made with other polymers over the last 3 decades. This is largely due to advances in reversible radical polymerization technology, which has made it possible to control chain length, monomer content and, to some extent, monomer sequence in a precise and reproducible manner (Guerassimoff et al., 2024).

Block copolymers, comprising a hydrophilic shell and a hydrophobic core, represent a promising class of carriers for drug delivery (Pottanam Chali and Ravoo, 2020). They are synthesized from blocks or fragments of monomers, and their assemblies are classified according to their structural characteristics, namely, linear, grafted, star-shaped or dense-armed. The use of block copolymers as carrier systems has been shown to extend the drug half-life and enhance the targeting of drugs derived from natural plants. Furthermore, they are frequently formulated as NPs, which typically have a diameter between 10 and 1,000 nm. The smaller particle size and the ability of NPs to load drugs may be crucial in the treatment of cancer, wound repair, and neuroendocrine diseases. Table 1 lists the drugs discussed in this paper and their paired polymer delivery methods.

**TABLE 1:** The applications of polymers in Alzheimer’s disease treatment.

Drug	Particle Size (nm)	Polymer’s carrier	Modifier	Major targets	References (first author, published year)
Curcumin	50–250	PEG-PLA	B6 peptite	Aβ Cascade Tau protein phosphorylati on	Fan et al. (2018)
Resveratrol	20	Chitosan	TG (TGNYKALHPHN)	Akt/ERK/GSK3β GLUT1/3 Gut microbiome	Yang et al. (2023)
Insulin	95.2 ± 19.0	Chitosan	Transfersulin	PI3K-Akt	Nojoki et al. (2022)
GV-971	NA	NA	NA	Gut microbiome/Gut-brain axis	Wang et al. (2019)
PLGA	100	NA	NA	Aβ Cascade Tau protein CatD	Anand et al. (2022) Wang et al. (2020a)
Ibuprofen	195.4	PEG-PLGA	NA	PCREB Neuroinflammation	Sánchez-López et al. (2017)
Memantine	152.6 ± 0.5	PEG-PLGA	NA	NMDAR	Sánchez-López et al. (2018)
Galanthamine	201 ± 1.2	Chitosan	NA	AchE	El-Ganainy et al. (2021)

PEG, polyethylene glycol; PLGA, poly (lactic-co-glycolic acid); PLA, poly (lactic acid); CatD, cathepsin D; pCREB, phosphorylated cyclic AMP response element-binding protein; NMDAR, N-methyl-D-aspartic acid receptor; AchE, acetylcholinesterase; NA, not available.

### 3.1 Poly (lactic-co-glycolic acid)

Poly (lactic-co-glycolic acid) (PLGA) has received extensive research attention because of its excellent biocompatibility and biodegradability (Hassan et al., 2024; Sonam Dongsar et al., 2024; Hadley et al., 2023; Hamadani et al., 2023). PLGAs are typically formed by ring-opening copolymerization of lactic acid (LA) and glycolic acid (GA), with the monomers linked by lipid bonds. The ratio of PLA to GA in the composition affects the hydrophobicity, size and rate of biodegradation (Schliecker et al., 2003). Incorporating GA reduces the polymer’s crystallinity while increasing the water absorption rate of the nanomaterial. Consequently, the degradation rate of PLGA can be finely tuned by adjusting the LA-to-GA ratio in the amorphous polymer. LA is crystalline, while GA exhibits more amorphous characteristics. A higher GA content shifts the ratio of crystalline to amorphous phases in PLGA particles toward the amorphous region, resulting in faster hydrolysis of the polymer particles. The most common ratio in the biomedical field is currently 50:50, because this ratio of polymers has the lowest crystallinity and the highest hydrophilicity, giving this ratio of PLGAs the fastest degradation rate (Lü et al., 2009; Allahyari and Mohit, 2016). This ratio of PLGA has been demonstrated to be particularly effective in facilitating drug delivery across the BBB. A comparison of the neuronal uptake of Cur, NPs-Cur 50:50, and NPs-Cur 65:35 revealed that SK-N-SH cells exhibited a higher uptake of NPs-Cur 50:50 than NPs-Cur 65:35 or free Cur (Djiokeng Paka et al., 2016).

The emulsification-solvent evaporation method is the most widely used technique due to its simplicity, uniform particle size, and high encapsulation efficiency. Thus, the method is particularly suitable for controlled drug release and targeted delivery systems. This method consists of two main stages, namely, emulsion preparation and solvent evaporation. During the emulsion preparation stage, emulsifiers can be formulated in various forms, such as water-in-oil (W/O), oil-in-water (O/W), water-in-oil-in-water (W/O/W), or solid-in-oil (S/O) emulsions (Sun et al., 2024). Among these, W/O/W emulsions are considered optimal for encapsulating water-soluble drugs such as peptides, proteins, and vaccines, while O/W emulsions are more suitable for encapsulating water-insoluble drugs such as paclitaxel and dexamethasone (Kias and Bodmeier, 2024; Hu et al., 2023; Jain, 2000). In addition to the emulsification-solvent evaporation method, other techniques such as spray-drying, nanoprecipitation, and phase separation are also employed for the preparation of NPs (Zhang et al., 2013; Makadia and Siegel, 2011; Mundargi et al., 2008).

Recent studies have revealed entirely new applications for PLGA, highlighting its potential not only as a drug delivery system but also as a therapeutic agent in its own right. Traditionally, PLGA has been used to transport drugs such as Cur, donepezil, and quercetin, but its intrinsic medicinal properties have often been overlooked. However, emerging research suggests that PLGA itself holds significant therapeutic value, including the ability to address pathological changes in AD (Fan et al., 2018; Jeon et al., 2019; Ji et al., 2023).

### 3.2 PEG

As mentioned earlier, PEG therapy has been approved for marketing as polymer-coupled drugs. Most PEGs used in clinical applications are covalently bonded to form PEG couplings with the target proteins (Grigoletto et al., 2016). PEG is the most commonly used polymer for drug modification. It is often conjugated with ligands by various methods, including physical absorption, chemical conjugation and molecular self-assembly. The relative complexity and cost of each method of synthesis differ (Shi et al., 2021). Although physical absorption offers the advantages of simplicity and ease of control, it requires a strong adsorption affinity between PEG or its derivatives and the substrate. Furthermore, this strategy still faces the challenge of low adsorption intensity (Kaur et al., 2008). The majority of PEGs are currently assembled with drugs through chemical coupling and molecular self-assembly. While the former entails covalent bonding between the drug and PEG, the latter typically occurs through nanoprecipitation or emulsification, enabling the synthesis of NPs with enhanced PEG coverage but requiring more sophisticated handling and conditions (Shi et al., 2020; Porte et al., 2019). Existing PEG applications are mainly in the form of diblock or triblock copolymers for drug delivery such as PEG-PLGA. Block copolymers show better release kinetics than PEG alone (Cheng et al., 2007). PEG has the ability to cover the lipophilic surface of PLGA, rendering the NPs hydrophilic. This reduces the uptake of the NPs by the liver, thereby prolonging their circulation time in the body and avoiding phagocytosis by the mononuclear phagocyte system (MPS). In addition, PEG can be conjugated to proteins to increase their molecular weight above the renal filtration threshold, thereby reducing renal clearance and significantly increasing the half-life of the drug in the bloodstream (in some cases by up to 20 times). Although it is now known that PEG-protein conjugation can mask active sites, several injectable PEG-protein conjugates are available (Gonçalves and Caliceti, 2024). However, researchers have identified several limitations of PEG in its current applications. These include immune reactions, which have been reported with intravenous injection, oral administration and topical application. High-molecular-weight PEG is nondegradable, and its synthesis process inevitably produces by-products (Knop et al., 2010).

### 3.3 Chitosan

Unlike the synthetic substances mentioned earlier, chitosan (CS) is a natural polymer mainly derived from natural crustaceans, namely, shrimps and crabs. CS is obtained from crustaceans after deacetylation of chitin. This polymer is easily modified at the C-2 position due to its special chemical structure, naturally carries cations that make it easier to be adsorbed by cells, has the ability to form ionic cross-links leading to the formation of stable complexes that release drugs slowly over a long period, thereby achieving controlled drug release, and has excellent biocompatibility and biodegradability (Younes and Rinaudo, 2015). The initial stage of the preparation of CS NPs entails the creation of a CS solution within an acidic milieu. The most common approach involves the use of a 1% acetic acid or hydrochloric acid buffer solution, followed by a pH adjustment in accordance with the specific derivative of CS under consideration (Mistry et al., 2009; Kaiser et al., 2015; Zhang et al., 2022). Once the CS solution is prepared, its combination with NPs can be achieved in two steps: 1) by adding the CS solution to preformed NPs, such as nanotubes, magnetic iron oxide NPs or liposomes, or 2) by incorporating the CS solution during the NP preparation process, which is commonly used for polymeric NPs, such as PLGA NPs mentioned earlier (Elkomy et al., 2022; Reshma et al., 2017). CS NPs are typically synthesized using a bottom-up ionic gelation method. This involves the preparation of a solution of an anionic crosslinker, such as sodium tripolyphosphate (TPP), and CS. These two reactants self-assemble into CS NPs through the action of electrostatic interactions between the positively charged amine groups of CS and the negatively charged polyanions (Baghdan et al., 2018). The biocompatibility and biodegradability of TPP make this method widely used in pharmaceutical preparation. In drug delivery, not only does CS improve the biocompatibility of drugs, but more importantly, it loosens the tight junctions of epithelial cells, thereby facilitating the paracellular transport of drugs across the epithelial barrier. Due to these same properties, CS has also been investigated for use in intranasal insulin delivery (Abbad et al., 2015; Sung et al., 2012). CS is now widely attempted to be used as a carrier for drug delivery (Hu and Luo, 2021; Imran et al., 2023).

## 4 Polymers as carriers for targeting AD drugs to improve bioavailability and delivery modalities

### 4.1 Oral or injection administration

Cur is considered an investigational drug in the treatment of AD. BACE-1 is one of the key enzymes for Aβ fiber production. How it cleaves APP to produce Aβ has been mentioned earlier. Chen et al. showed a significant reduction in BACE1 in mice with simulated AD after gavage with Cur’s saline (15 mg/mL and 30 mg/mL), with no change in the expression level of APP in the mice tested (Zheng K. et al., 2017). This result illustrates the great potential value of Cur in AD therapy. Its poor bioavailability, short *in vivo* half-life and difficulty in passing the BBB have been hindering the further application of this material. However, polymeric NP complexes offer more possibilities to Cur. B6 peptide is known to target Tfr in some capillary endothelial cells and neurons in the brain and can enter the CNS via RMC (Liu et al., 2013). On this basis, Cur-loaded PLA-PEG NPs conjugated with B6 (PEG-PLA-B6/Cur) were administered to APP/PS1A1 transgenic mice. In addition to reduced aggregation of Aβ protein and phosphorylation of tau protein, protein analyses also revealed the inhibition of BACE1, APP and PS1. PEG-PLA-B6/Cur also showed a better slow release of the drug *in vitro* than free Cur (Fan et al., 2018).

In recent years, a growing number of researchers have tried to target neuroinflammation to treat AD. However, due to problems such as incomplete release and poor bioavailability, it is imperative to improve drug delivery carriers (Bonabello et al., 2003; Kaehler et al., 2003). Dexibuprofen (DXI) was used to synthesize PLGA surrounded by PEG chains (DXI-PLGA-PEG nanospheres (NSs)) with a larger surface area and adhesion. Of note when using DXI-PLGA - PEG NSs in mice, the expression of p-CREB, a protein related to synaptic plasticity and memory increased (Benito and Barco, 2010). Moreover, a reduction in fibrous plaques was observed in mice treated with NSs. The authors noted that this may be due to the ability of DXI to inhibit the associated inflammatory response, while PEG can reduce amyloid-insoluble plaques by helping NSs to cross the BBB through endocytosis. The increased expression of p-CREB may be attributed to this as well. The weight of the gastric mucosa in the NS group was second only to that of the untreated mice, suggesting that loading the drugs with NPs attenuated the gastric damage caused by the free drugs (Sánchez-López et al., 2017). DXI-loaded NSs overcome many of the side effects of free drugs and can be turned into a safe strategy for AD prevention.

Polyethylene glocalization of NPs prevents them from being recognized by the reticuloendothelial system and reduces their rate of clearance by decreasing the interaction with mucins (Knop et al., 2010; Griffiths et al., 2015). PEG is a paired with a marketed drugs to optimize their slow release profile. Memantine (MEM) is an NMDA receptor inhibitor approved for AD treatment, NMDAR is present on the postsynaptic membrane and its hyperactivation leading to a large inward flow of Ca^2+^ is considered one of the main causes of synaptic failure in AD patients (Johnson and Kotermanski, 2006). In animal studies, transgenic APPswe/PS1dE9 mice were administered with MEM-loaded PEG-PLGA NPs, and the results showed a slow release of NPs using this delivery system; furthermore, a more pronounced reduction in amyloid plaques was observed in the brains of mice that received MEM-loaded PEG-PLGA NPs than in those that received free drug solutions. A more direct path to the platform was also demonstrated in the Morris water maze test (Sánchez-López et al., 2018). MEM-PEG-PLGA is a more promising alternative to free drugs.

Among the therapies targeting BACE-1, attempts have also been made to reduce the expression of BACE-1 by delivering siRNAs that target BACE-1 effectively and specifically to neurons (Singer et al., 2005). Conventional adenoviral or lentiviral vector-based drug delivery methods face great challenges due to their insecurity and inconvenience. Researchers have experimented with the use of nanocarriers for drug delivery. The cationic polymer poly (2-(N,N-dimethylamino)ethyl methacrylate) (PDMAEMA) was used for drug loading and to prevent unwanted interactions with negatively charged DNA, as well as to avoid blood agglutination. To enhance its stability, PEG was conjugated to PDMAEMA. The PEG-PDMAEMA conjugate was subsequently identified as an optimal vector for siRNA delivery, due to its low toxicity and high transfection efficiency (Qian et al., 2013; van Steenis et al., 2003). To help NPs cross the BBB and target amyloid plaques in the brain, CGN peptide (d-CGNHPHLAKYNGT) and QSH peptide were further synthesized. Both have good affinity for brain capillary endothelial cells and Aβ (Zhang et al., 2014). The hybrid complex CQ/siRNA, composed of 25% MPEG-PDMAEMA, 50% CGN-PEG-PDMAEMA and 25% QSH-PEG-PDMAEMA, enters the cell via lattice protein-mediated endocytosis and subsequently escapes from the lysosome to act on the mRNA (Zheng X. et al., 2017). It is unclear how NPs escape the lysosome, and the most widely accepted theory is a proton sponge effect sowing to the cationic PEG-PDMAEMA (Won et al., 2009). Another study replaced QSH with the neuron-targeting ligand Tet1 on the hemolytic effects of the drugs and found that PEG-PDMAEMA effectively prevented erythrocyte interactions and aggregation, which the authors indicated was due to the steric hindrance and charge shielding achieved by PEG chain on the surface of the complexes. The same study also investigated the effect of siRNA against BACE-1 on the expression of myelin basic protein (MBP, 14–21.5 kDa), as myelin dysplasia was found in mice with deletion of the BACE1 gene, and western blotting showed no significant adverse effect of CT/siRNA on myelin sheaths (Wang P. et al., 2018). The study has presented compelling evidence that PEG-PDMAEMA carriers can effectively deliver siRNA across the BBB and be used in the treatment of AD.

CS has been shown to increase the stability of bioactive molecules exposed to the gastrointestinal tract for oral administration. Res was attempted to treat AD via the brain-gut axis to increase Res activity in the organism. CS was cross-linked with sodium TPP to encapsulate poorly water-soluble Res to enhance its solubility and stability. (Wu et al., 2017). The subsequent modification of the brain-targeting peptide (TG: TGNYKALHPHNG) resulted in the synthesis of TG-Res-CS/TPP-NPs. The CS-modified drug was subjected to *in vitro* simulation of gastric and intestinal fluids and it was found to have slower release profiles and higher stability than Res (Yang et al., 2023).

### 4.2 Intranasal administration

CS can deliver drugs to the CNS bypassing the BBB through intranasal administration, thereby reducing the side effects of drugs in peripheral tissues or organs. This is because CS can open the tight junctions between epithelial cells by inhibiting PKC activity and transferring proteins such as ZO-1 therein from the cell membrane into the cytoplasm (Casettari and Illum, 2014; Smith et al., 2005). CS has a pH of 6.5, which makes it positively charged in the nasal cavity with a pH between 5.5 and 6.5. This leads to a longer retention time of CS-based drugs in the nasal cavity (Kazemi Shariat Panahi et al., 2023). Compared with traditional oral drug delivery, intranasal drug delivery can increase the bioavailability of encapsulated drugs in the brain by transcellular or paracellular pathways that cross the nasal epithelium to deliver drugs directly to the CNS via the olfactory bundle or trigeminal nerve (Lee and Minko, 2021; Jamshidnejad-Tosaramandani et al., 2024).

Insulin therapy is a novel treatment modality for AD, and there is evidence that diet-induced obesity and insulin dysregulation are closely linked to a range of pathological changes such as Aβ amyloid deposition and Tau protein aggregation in AD (Kellar and Craft, 2020; Flores-Cordero et al., 2022). Deficiency of GLUT1 and glucose transporter protein 3 in the BBB has been observed in AD patients. Insulin delivery using intranasal administration has been tried for the treatment of memory disorders, leading to enhanced memory in mice (Mao et al., 2016). In recent years, there have been clinical trials of intranasal administration of insulin, but because of the limitations of the dose, the effect is not particularly satisfactory, which puts forward higher requirements for the insulin delivery device (Shibata et al., 2000). To further improve the bioavailability of insulin by increasing its intranasal residence time, CS has been attempted as a drug carrier for insulin drug delivery. Using the membrane hydration method, researchers have achieved success in loading insulin into transfersome vesicles, which are ultra-deformable vesicles containing phospholipids and an edge activator (EA) (Opatha et al., 2020). The prepared transinsulin was added to 0.6% CS formamide salt buffer for hydration, and a CS-Transfersulin (CTI) with a CS film attached to the surface was prepared. The average size of the finished CTI was 137.9 ± 28.2 nm. In addition, 5-isothiocyanate (FITC) was added for staining, and fluorescence imaging showed that FITC-CTI conjugated with CS gradually entered the brain and entered the nasal cavity and olfactory vesicles in a short period of time. However, FITC-INS gradually dispersed to the peripheral organs after a short period (15 min). After 4 h, the fluorescence in other internal parts disappeared, and the fluorescence intensity of the lower organs increased. Morphological improvement of pyramidal cells in the highest hippocampus area after CTI treatment was observed in pathological tissue sections (Nojoki et al., 2022), suggesting a better brain-targeted delivery of insulin therapy using CS loading than free insulin.

Intranasal administration has the added advantage of avoiding drug contact with peripheral tissues. Galantamine, a marketed therapeutic drug for AD, loaded on CS, is an acetylcholinesterase (AchE) inhibitor. In the past, galantamine was commonly administered orally in the clinical setting, but in addition to the corresponding therapeutic effects, patients experienced adverse effects such as nausea, vomiting, diarrhea, and weight loss, which were presumed to be caused by the nonspecific binding of galantamine to peripheral AchE (Prvulovic et al., 2010). Intranasal administration provides a viable alternative mode of drug delivery. GH/CS complex NPs (CX-NPs) have been reported to show good biocompatibility in in vivo experiments (Duan et al., 2024). In mice with scopolamine-induced AD, CS loaded with galantamine was administered intranasally, intravenously and orally. Of the three modes of administration, the rats administered intranasally had the lowest plasma concentrations and the highest brain concentrations (El-Ganainy et al., 2021). It is important to note that care needs to be taken in the incorporation of other groups to alter the properties of CS. Researchers attempting to add alginate to drugs to improve the solubility of CS in environments with pH > 6.5 found that CS loaded with galantamine alone showed a slower release profile than GH-loaded CS/alginate NPs in in vitro experiments at pH = 7.2 where a gradual release of the drug was observed after 8 h, suggesting that alginate and CS shortened the release time of the drug (Georgieva et al., 2023). The above experiments demonstrated the delivery efficiency and slow-release profile as well as the excellent biocompatibility and biodegradability of the CS-loaded different drugs for intranasal administration.

### 4.3 Limitations of polymeric carriers in AD therapy

Although polymeric drugs, led by PEG, PLGA and CS, have made great progress in recent years in a variety of anti-AD drug delivery systems, most of them are still at the stage of animal testing where the combination of polymer and drug makes the metabolism kinetics of the drug *in vivo* more complex. For example, compared with traditional drugs, the ratio of polymer to drug substrate, molecular weight, degree of crystallinity, particle size and even the preparation process all affect the release of the drug in the bio-endogenous environment (Sun et al., 2024). This adds to the complexity of polymeric drugs in drug discovery and preparation. It has been shown that drug type, drug distribution and drug loading rate affect the drug release behavior of drug-loaded PLGA NPs, and that uniform drug distribution within the polymer matrix can lead to an early burst of drug release. To address this issue, researchers have designed a drug gradient distribution, with a higher concentration at the core and a lower concentration at the periphery, allowing for more stable drug release (Kakish et al., 2002). The metabolic pathways of polymeric drugs in the body remain unclear. To date, applications of PLGA-PEG block copolymers in cancer treatment have shown that the metabolic pathway of PEG is still unknown. However, no long-term studies have yet determined whether PEG can be cleared from the body, where it accumulates, or what effects it may have at the sites of accumulation (Knop et al., 2010). A recent review has shown that patients receiving PEG-based treatments experience prolonged neutropenia and coagulation dysfunction, with hepatotoxicity being significantly associated with PEGylated products compared with non-PEGylated counterparts. The study has also highlighted other notable adverse events associated with PEG-based therapies, such as hypersensitivity reactions and an overall increase in the risk of infection (Lee et al., 2024). CS also faces comparable challenges in the management of AD, particularly during intranasal administration. Further experimentation is required to confirm the safety of CS within the body, particularly within the CNS.

## 5 Native polymers as AD drugs

### 5.1 PLGA

According to Kar et al., PLGA NPs can protect neurons subjected to Aβ aggregation without being functionalized by any drug, and this material reduces the toxic effects produced by Aβ on the cells (Wang Y. et al., 2020). These researchers focused on cathepsin D (CatD), a cathepsin present in normal cellular lysosomes. Abnormal release of CatD from lysosomes led to the release of cytochrome c from mitochondria in the presence of dATP/ATP, which was capable of activating caspase-9, followed by activation of the caspase-3 apoptotic pathway (Wang F. et al., 2018; Repnik et al., 2012; Stoka et al., 2016). By observing defective Aβ catabolism in neurons of CatD-knockout mice, another study from the same period concluded that CatD was phagocytic toward Aβ proteins under physiological conditions (Suire et al., 2020). Given that the cytotoxic effects of CatD were observed by Kar et al., in 5XFAD mice, and that excess intracellular Aβ led to lysosomal damage, the source of this discrepancy may lie in whether or not the integrity of the lysosome is compromised. Once the permeability of the lysosomal membrane is increased, CatD is abnormally released into the cytoplasm inducing the apoptotic pathway. Several studies have been conducted on CatD for disease cure (Marques et al., 2020; Hossain et al., 2021). Lysotracker is a weakly basic stain that labels cellular compartments in cells with low pH (Wolfe et al., 2013). A reduction in neuronal death was observed in Aβ_1-42_-treated neurons exposed to 200 μg/mL PLGA for 12 h and this treatment reversed the diffuse staining of Lysotracker in Aβ mice, with most of the natural PLGA entering the neuronal cell via a lattice protein/vesicle-dependent pathway and internalizing through macrophage action. Subsequently, the PLGA was transported to the lysosomes via endosomes (Wang Y. et al., 2020). Another report indicated that PLGA could restore the pH of lysosomes damaged by alkalinization and to some extent improve lysosomal function (Baltazar et al., 2012). Using immunoblotting, a significant reduction in carbonyl levels in Aβ neurons was observed and it was hypothesized that the protective effect of PLGA on neurons may be mediated by a reduction in reactive [oxygen species (ROS)] (Wang Y. et al., 2020). In addition, it has been found that PLGA could reduce the expression of APP, α-secretase and β-secretase, however, further experiments are still needed to elucidate the specific mechanism by which PLGA regulates the transcription/processing of APP (Wu et al., 2022).

**FIGURE 3:**
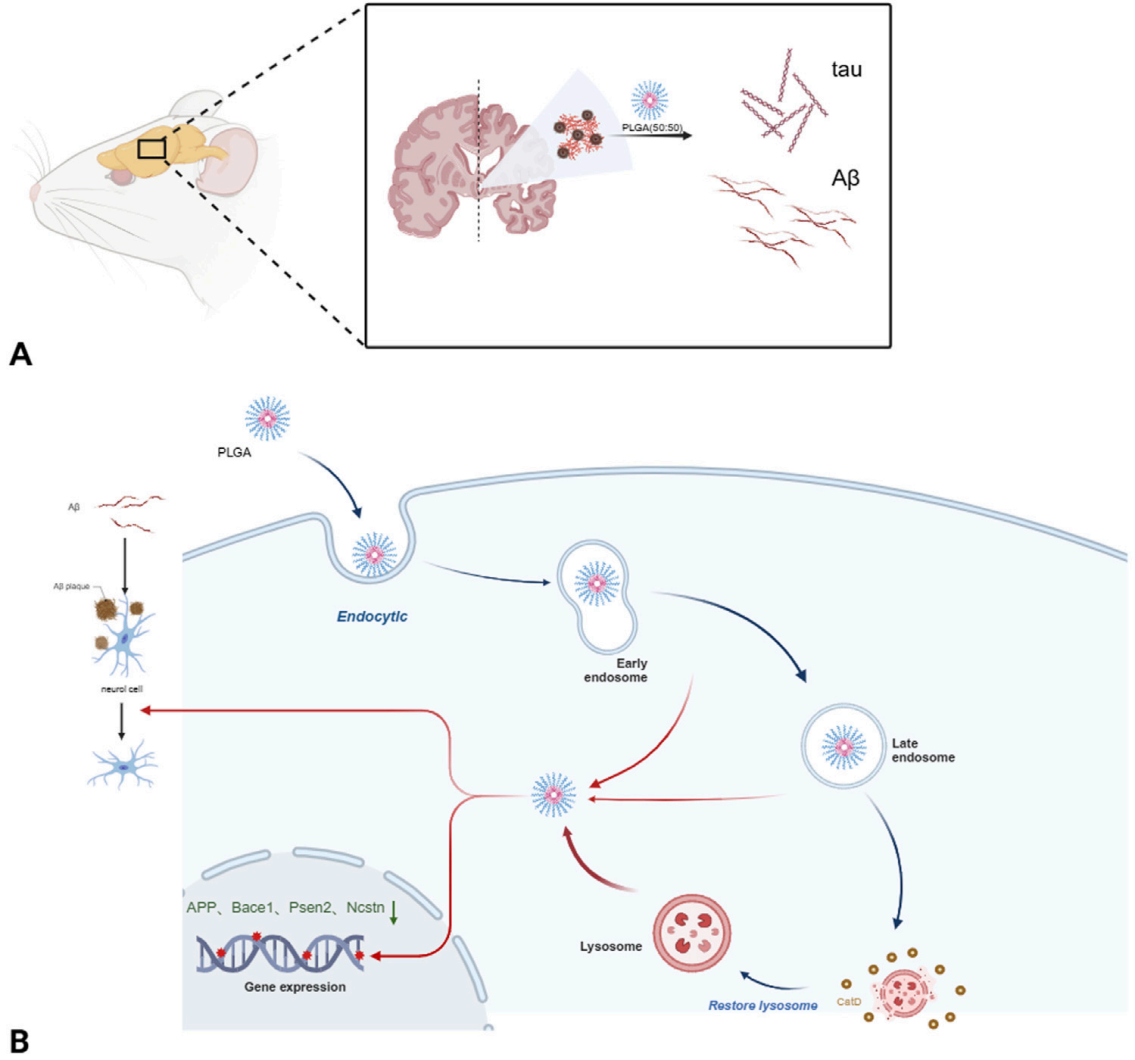
Specific mechanisms of PLGA in the treatment of Alzheimer’s disease. **(A)** PLGA inhibits Aβ_1-42_ and tau protein aggregation. **(B)** PLGA is present in early and late endosomes and lysosomes and restores damaged lysosomes by internalisation upon cell entry and reduces the expression of genes involved in APP processing such as APP, Bace1, Psen2, and Ncstn.

PLGA not only protects against intracellular toxicity caused by Aβ, but it also prevents Aβ aggregation and depolymerizes already aggregated Aβ. A significant reduction in the number of Aβ aggregates was obtained in neurons treated with PLGA. The results of fluorescent labeling showed PLGA-induced depolymerization of Aβ in a dose-dependent manner, suggesting that the direct interaction between the two is the basis for the unraveling of the chains, as shown in Figure 3. Spectroscopic studies, biochemical analyses and molecular dynamics simulations describing the interaction of PLGA NPs predominantly with the hydrophobic structural domains of Aβ_1–42_ corroborate this conclusion (Paul et al., 2022). By adjusting the proportion of PLGA to GA, an equimolar PLGA of 75:25 isomers, 50 μM LA, 50 μM GA or a mixture of 50 μM LA and GA was found not to change the aggregation of Aβ illustrating the specificity of the results (Anand et al., 2022). The above experiments demonstrate the promise of the therapeutic effects of PLGA when used alone as a drug for the range of impairments caused by Aβ in AD. Recent reports have indicated that PLGA may have the potential to inhibit the formation of neurofibrillary tangles associated with tau phosphorylation (Paul et al., 2024).

### 5.2 CS oligosaccharide

CS oligosaccharide (COS) is a hydrolyzed product of CS. Because of its lower molecular weight, COS has higher solubility and lower viscosity under physiological conditions than CS (Zhou et al., 2010). COS and its derivatives have been used in a large number of biomedical and pharmaceutical applications, and most of the derivatives developed for COS are directed toward the hydroxyl and/or amine/acetamide groups (Naveed et al., 2019). Previous studies have shown that COS has an outstanding role in against oxidative stress, inhibits β-secretase and exerts anti-inflammatory effects (Ouyang et al., 2017). Sun et al. used COS in rats treated with Aβ_1-42_, and three doses (200, 400, and 800 mg/kg) were found to reduce neuronal death in the Aβ_1-42_-exposed rats (Jia et al., 2016). Modification of COS using different groups to obtain caffeic acid conjugated-COS enhanced its inhibition of β-secretase (Eom et al., 2013). Neuroinflammation is also one of the important therapeutic targets for AD. Peracetylated COS (PACOS) may significantly affect the PI3K-Akt signaling pathway and cell proliferation-related pathways, and alleviate the aggregation of Aβ protein in a dose-dependent manner. Improved the repair of β-amyloid-induced cognitive deficits in rats. After the same PACOS treatment (25, 50, 100 mg/kg), compared with untreated rats, the phosphorylated tau protein levels were significantly different to approximately 1,660, 1,500, and 1,350 pg/mL (P < 0.05), and a significant decrease in the levels of inflammatory factors TNF-α and IL-6 was observed (Hao et al., 2023). COS also inhibited the MAPK and NF-κB pathways by upregulating heat shock protein 70 (HSP 70) and downregulating HSP 90, thereby attenuating oxidative stress in neurons and preventing subsequent apoptosis (Joodi et al., 2011). In summary, COS is another drug that may be used in the treatment of AD.

### 5.3 GV-971

**FIGURE 4:**
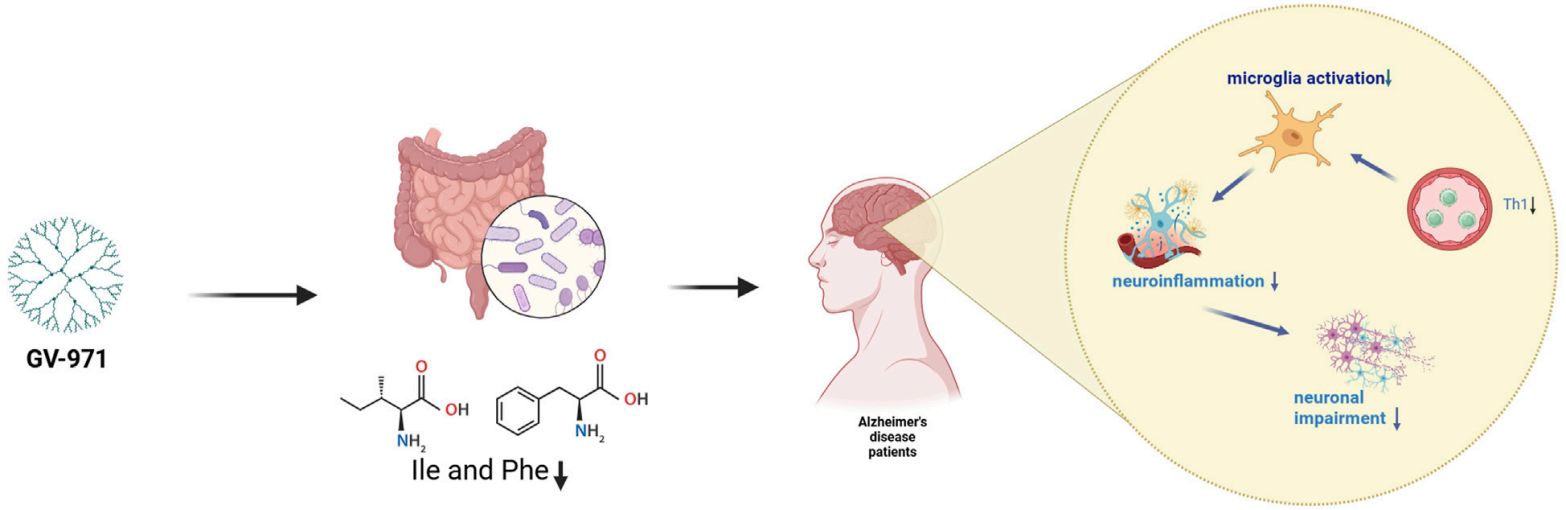
GV-971 relieves neuroinflammation in the middle brain GV-971 further reduces Th1 cell activation by modulating amino acid metabolism in the gut flora, ultimately reducing neuroinflammation.

Another oligosaccharide, GV-971, was approved for marketing in China in November 2019. It is a natural oligomer with a molecular weight of around 1,000 Da and targets the gut flora to alleviate AD neuroinflammation. This substance is derived from natural alginate, and is produced by depolymerizing propylene glycol alginate sodium sulfate followed by oxidation, leaving the carboxyl group at the reduced end (Gao et al., 2019). It can cross the BBB via GLUT1 carrier protein translocation or the paracellular pathway (Lu et al., 2022; Wang et al., 2019). As AD progresses, Aβ protein and tau phosphorylation may lead to disturbed gut metabolism in patients, which in turn causes an inflammatory response and brain infiltration by immune cells (Alkasir et al., 2017). GV-971 can reduce inflammatory responses by normalizing the disordered gut metabolism. Specifically, by regulating the metabolism of phenylalanine and isoleucine in the intestinal flora, inhibition of phenylalanine-induced Th1 cell proliferation further reduces microglial cell activation, as shown in Figure 4 (Wang et al., 2019). In the 5XFAD experiment, the reduction in Aβ load in the brain of male mice with administered different doses of GV-971 (40 mg/kg, 80 mg/kg, 160 mg/kg) was most pronounced in the 80 mg/kg group. Interestingly, this therapeutic effect was only found in male mice. The same sex-specificity was found in studies on neuroinflammation via astrocytes and microglia, which is consistent with previous therapeutic results using antibiotic cocktail (ABX), Both ABX and GV-971 target the gut microbiota, and speculation that this phenomenon may be due to a variety of complex causes such as ovarian hormones and other causes (Lopez-Lee et al., 2024; Dodiya et al., 2019; Bosch et al., 2024). However, gender specificity has not been reported in previously completed clinical trials. No amyloid or tau protein biomarkers have been used in any of the currently completed clinical trials on GV-971. The treatment effect in question was primarily reflected in AD Cooperative Study-Activities of Daily Living (ADAS-cog12), Neuropsychiatric Inventory and CIBIC-plus responses. All three scales showed significant improvement compared with the placebo group. Patient compliance with GV-971 in the trial was good, with no large-scale adverse reactions reported (Xiao et al., 2021; Wang T. et al., 2020; Chen et al., 2024). However, the bioavailability of GV-971 does not appear to be optimal. In the experimental pharmacokinetic study of GV-971, it was found that the bioavailability of GV-971 was very low in rats (0.6%–1.6%) and dogs (4.5%–9.3%), and most of the drug that had been taken into the bloodstream was rapidly metabolized by the kidneys and excreted in the urine. The rest was likely absorbed by the intestinal flora as nutrients and then eliminated in the feces (Lu et al., 2022).

Although GV-971 has been approved for marketing, there are many controversies concerning its use. There is no apparent AD-relevant molecular target, bioavailability is low, understanding of the brain–gut axis is still in its infancy, and many questions about GV-971 have been raised (Lu et al., 2022; Yeo-Teh and Tang, 2023). Furthermore, thus far clinical trials of GV-971 have not included AD-related biomarkers as part of the subject selection criteria, because amyloid positron emission tomography was not widely available in China at the time the trial was planned and initiated. However, AD biomarkers were included as part of the diagnostic criteria in a clinical trial initiated in the USA. The results of this trial, which is scheduled to be completed in 2026 (NCT04520412), may fill a gap in the knowledge of the effects of GV-971 on amyloid plaques in humans. We expect that GV-971 will be able to go further to the international market through this trial and bring benefits to more patients.

## 6 Conclusion and future prospects

To date, polymers have demonstrated the capacity to address numerous challenges that traditional drugs are unable to surmount in the treatment of AD, thereby illustrating their prospective value in the domain of medicine. Researchers have modified the release profiles and half-lives of various pharmaceuticals within the body by altering particle size, material distribution, polymer molecular weight, and shape.

Given the numerous advantages of polymers and polymer drugs that have been outlined earlier, many polymers have progressed to clinical trials. In the future, the development of polymeric drugs for AD will have to overcome the following challenges: 1) It is unclear whether the metabolic destination of polymer drugs in the body is consistent with that of pure polymers. If these large molecules are not filtered by the glomeruli, it is necessary to determine where they will accumulate in the body, how they will be cleared, and what impact they will have on the body. Further long-term observations are required to determine the toxic effects of polymers in the body. 2) The reason for the additional permeability of the BBB to of polymer drugs, the mechanism of their passage through the BBB, and the subsequent metabolism of these drugs in the CNS must be elucidated. 3) siRNA therapy is already being used in clinical trials to treat cancer, transthyretin amyloidosis and primary hyperoxaluria type I (Zatsepin et al., 2016). However, the annual cost of this therapy can be in the hundreds of thousands of dollars per patient, a problem shared by other drugs. Given the large number of people with AD this high cost could put treatment out of reach for most families.

With regard to native polymers, the number of fundamental studies pertaining to their mechanisms and pharmacokinetic processes remains inadequate, despite the considerable advantages they offer, including straightforward and cost-effective preparation methods. It is recommended that further research be conducted by additional researchers to gain a more comprehensive understanding of the role of these substances in AD therapy. This should include studies on bioavailability and cytotoxicity when the substances are administered orally or by injection.

## References

[B1] Abbad S. Zhang Z. Waddad A. Y. Munyendo W. L. Lv H. Zhou J. (2015). Chitosan-modified cationic amino acid nanoparticles as a novel oral delivery system for insulin. J. Biomed. Nanotechnol. 11 (3), 486–499. 10.1166/jbn.2015.1924 26307831

[B2] Agrahari V. Agrahari V. (2018). Advances and applications of block-copolymer-based nanoformulations. Drug Discov. Today 23 (5), 1139–1151. 10.1016/j.drudis.2018.03.004 29551456

[B3] Alkasir R. Li J. Li X. Jin M. Zhu B. (2017). Human gut microbiota: the links with dementia development. Protein Cell. 8 (2), 90–102. 10.1007/s13238-016-0338-6 27866330 PMC5291774

[B4] Allahyari M. Mohit E. (2016). Peptide/protein vaccine delivery system based on PLGA particles. Hum. Vaccin Immunother. 12 (3), 806–828. 10.1080/21645515.2015.1102804 26513024 PMC4964737

[B5] Anand B. Wu Q. Nakhaei-Nejad M. Karthivashan G. Dorosh L. Amidian S. (2022). Significance of native PLGA nanoparticles in the treatment of Alzheimer's disease pathology. Bioact. Mater 17, 506–525. 10.1016/j.bioactmat.2022.05.030 36330076 PMC9614411

[B6] Andronie-Cioara F. L. Ardelean A. I. Nistor-Cseppento C. D. Jurcau A. Jurcau M. C. Pascalau N. (2023). Molecular mechanisms of neuroinflammation in aging and Alzheimer's disease progression. Int. J. Mol. Sci. 24 (3), 1869. 10.3390/ijms24031869 36768235 PMC9915182

[B7] Aston-Jones G. Rogers J. Shaver R. D. Dinan T. G. Moss D. E. (1985). Age-impaired impulse flow from nucleus basalis to cortex. Nature 318 (6045), 462–464. 10.1038/318462a0 4069217

[B8] Babaei P. (2021). NMDA and AMPA receptors dysregulation in Alzheimer's disease. Eur. J. Pharmacol. 908, 174310. 10.1016/j.ejphar.2021.174310 34265291

[B9] Baghdan E. Pinnapireddy S. R. Strehlow B. Engelhardt K. H. Schäfer J. Bakowsky U. (2018). Lipid coated chitosan-DNA nanoparticles for enhanced gene delivery. Int. J. Pharm. 535 (1-2), 473–479. 10.1016/j.ijpharm.2017.11.045 29175439

[B10] Baltazar G. C. Guha S. Lu W. Lim J. Boesze-Battaglia K. Laties A. M. (2012). Acidic nanoparticles are trafficked to lysosomes and restore an acidic lysosomal pH and degradative function to compromised ARPE-19 cells. PLoS One 7 (12), e49635. 10.1371/journal.pone.0049635 23272048 PMC3525582

[B11] Benito E. Barco A. (2010). CREB's control of intrinsic and synaptic plasticity: implications for CREB-dependent memory models. Trends Neurosci. 33 (5), 230–240. 10.1016/j.tins.2010.02.001 20223527

[B12] Birks J. S. Grimley Evans J. (2015). Rivastigmine for Alzheimer's disease. Cochrane Database Syst. Rev. (4), Cd001191. 10.1002/14651858.CD001191.pub3 25858345

[B13] Birks J. S. Harvey R. J. (2018). Donepezil for dementia due to Alzheimer's disease. Cochrane Database Syst. Rev. 6 (6), Cd001190. 10.1002/14651858.CD001190.pub3 12917900

[B14] Bonabello A. Galmozzi M. R. Canaparo R. Isaia G. C. Serpe L. Muntoni E. (2003). Dexibuprofen (S+-isomer ibuprofen) reduces gastric damage and improves analgesic and antiinflammatory effects in rodents. Anesth. Analg. 97 (2), 402–408. 10.1213/01.Ane.0000073349.04610.42 12873925

[B15] Bosch M. E. Dodiya H. B. Michalkiewicz J. Lee C. Shaik S. M. Weigle I. Q. (2024). Sodium oligomannate alters gut microbiota, reduces cerebral amyloidosis and reactive microglia in a sex-specific manner. Mol. Neurodegener. 19 (1), 18. 10.1186/s13024-023-00700-w 38365827 PMC10874048

[B16] Cabral H. Miyata K. Osada K. Kataoka K. (2018). Block copolymer micelles in nanomedicine applications. Chem. Rev. 118 (14), 6844–6892. 10.1021/acs.chemrev.8b00199 29957926

[B17] Casettari L. Illum L. (2014). Chitosan in nasal delivery systems for therapeutic drugs. J. Control Release 190, 189–200. 10.1016/j.jconrel.2014.05.003 24818769

[B18] Chen Y. Lai M. Tao M. (2024). Evaluating the efficacy and safety of Alzheimer's disease drugs: a meta-analysis and systematic review. Med. Baltim. 103 (16), e37799. 10.1097/md.0000000000037799 PMC11029996 38640313

[B19] Cheng J. Teply B. A. Sherifi I. Sung J. Luther G. Gu F. X. (2007). Formulation of functionalized PLGA-PEG nanoparticles for *in vivo* targeted drug delivery. Biomaterials 28 (5), 869–876. 10.1016/j.biomaterials.2006.09.047 17055572 PMC2925222

[B20] Djiokeng Paka G. Doggui S. Zaghmi A. Safar R. Dao L. Reisch A. (2016). Neuronal uptake and neuroprotective properties of curcumin-loaded nanoparticles on SK-N-sh cell line: role of poly(lactide-co-glycolide) polymeric matrix composition. Mol. Pharm. 13 (2), 391–403. 10.1021/acs.molpharmaceut.5b00611 26618861

[B21] Dodiya H. B. Kuntz T. Shaik S. M. Baufeld C. Leibowitz J. Zhang X. (2019). Sex-specific effects of microbiome perturbations on cerebral Aβ amyloidosis and microglia phenotypes. J. Exp. Med. 216 (7), 1542–1560. 10.1084/jem.20182386 31097468 PMC6605759

[B22] Duan L. H. Li L. I. Wang C. B. Liu Q. Q. Zhang X. Wu Z. Z. (2024). Brain targeting efficacy of novel drug delivery system in the treatment of Alzheimer's disease. Eur. Rev. Med. Pharmacol. Sci. 28 (13), 3892–3904. 10.26355/eurrev_202407_36521 39012229

[B23] Ekladious I. Colson Y. L. Grinstaff M. W. (2019). Polymer-drug conjugate therapeutics: advances, insights and prospects. Nat. Rev. Drug Discov. 18 (4), 273–294. 10.1038/s41573-018-0005-0 30542076 PMC12032968

[B24] El-Ganainy S. O. Gowayed M. A. Agami M. Mohamed P. Belal M. Farid R. M. (2021). Galantamine nanoparticles outperform oral galantamine in an Alzheimer's rat model: pharmacokinetics and pharmacodynamics. Nanomedicine (Lond). 16 (15), 1281–1296. 10.2217/nnm-2021-0051 34013783

[B25] Elkomy M. H. Ali A. A. Eid H. M. (2022). Chitosan on the surface of nanoparticles for enhanced drug delivery: a comprehensive review. J. Control Release 351, 923–940. 10.1016/j.jconrel.2022.10.005 36216174

[B26] Eom T. K. Ryu B. Lee J. K. Byun H. G. Park S. J. Kim S. K. (2013). β-secretase inhibitory activity of phenolic acid conjugated chitooligosaccharides. J. Enzyme Inhib. Med. Chem. 28 (1), 214–217. 10.3109/14756366.2011.629197 22424182

[B27] Fan S. Zheng Y. Liu X. Fang W. Chen X. Liao W. (2018). Curcumin-loaded PLGA-PEG nanoparticles conjugated with B6 peptide for potential use in Alzheimer's disease. Drug Deliv. 25 (1), 1091–1102. 10.1080/10717544.2018.1461955 30107760 PMC6116673

[B28] Fani G. Mannini B. Vecchi G. Cascella R. Cecchi C. Dobson C. M. (2021). Aβ oligomers dysregulate calcium homeostasis by mechanosensitive activation of AMPA and NMDA receptors. ACS Chem. Neurosci. 12 (4), 766–781. 10.1021/acschemneuro.0c00811 33538575 PMC7898266

[B29] Feng H. Lu X. Wang W. Kang N. G. Mays J. W. (2017). Block copolymers: synthesis, self-assembly, and applications. Polym. (Basel) 9 (10), 494. 10.3390/polym9100494 PMC6418972 30965798

[B30] Flores-Cordero J. A. Pérez-Pérez A. Jiménez-Cortegana C. Alba G. Flores-Barragán A. Sánchez-Margalet V. (2022). Obesity as a risk factor for dementia and Alzheimer's disease: the role of leptin. Int. J. Mol. Sci. 23 (9), 5202. 10.3390/ijms23095202 35563589 PMC9099768

[B31] Gao Y. Zhang L. Jiao W. (2019). Marine glycan-derived therapeutics in China. Prog. Mol. Biol. Transl. Sci. 163, 113–134. 10.1016/bs.pmbts.2019.02.006 31030745

[B32] Georgieva D. Nikolova D. Vassileva E. Kostova B. (2023). Chitosan-based nanoparticles for targeted nasal galantamine delivery as a promising tool in Alzheimer's disease therapy. Pharmaceutics 15 (3), 829. 10.3390/pharmaceutics15030829 36986689 PMC10056147

[B33] Gonçalves J. Caliceti P. (2024). Optimizing pharmacological and immunological properties of therapeutic proteins through PEGylation: investigating key parameters and their impact. Drug Des. Devel Ther. 18, 5041–5062. 10.2147/dddt.S481420 PMC11552514 39529843

[B34] Greenamyre J. T. Young A. B. (1989). Excitatory amino acids and Alzheimer's disease. Neurobiol. Aging 10 (5), 593–602. 10.1016/0197-4580(89)90143-7 2554168

[B35] Griffiths P. C. Cattoz B. Ibrahim M. S. Anuonye J. C. (2015). Probing the interaction of nanoparticles with mucin for drug delivery applications using dynamic light scattering. Eur. J. Pharm. Biopharm. 97 (Pt A), 218–222. 10.1016/j.ejpb.2015.05.004 25986588

[B36] Grigoletto A. Maso K. Mero A. Rosato A. Schiavon O. Pasut G. (2016). Drug and protein delivery by polymer conjugation. J. Drug Deliv. Sci. Technol. 32, 132–141. 10.1016/j.jddst.2015.08.006

[B37] Guerassimoff L. Ferrere M. Bossion A. Nicolas J. (2024). Stimuli-sensitive polymer prodrug nanocarriers by reversible-deactivation radical polymerization. Chem. Soc. Rev. 53 (12), 6511–6567. 10.1039/d2cs01060g 38775004 PMC11181997

[B38] Hadley P. Chen Y. Cline L. Han Z. Tang Q. Huang X. (2023). Precise surface functionalization of PLGA particles for human T cell modulation. Nat. Protoc. 18 (11), 3289–3321. 10.1038/s41596-023-00887-8 37853157 PMC10775953

[B39] Hamadani C. M. Dasanayake G. S. Gorniak M. E. Pride M. C. Monroe W. Chism C. M. (2023). Development of ionic liquid-coated PLGA nanoparticles for applications in intravenous drug delivery. Nat. Protoc. 18 (8), 2509–2557. 10.1038/s41596-023-00843-6 37468651

[B40] Hao C. Han M. Wang W. Yang C. Wang J. Guo Y. (2023). The neuroprotective effects of peracetylated chitosan oligosaccharides against β-amyloid-induced cognitive deficits in rats. Mar. Life Sci. Technol. 5 (2), 211–222. 10.1007/s42995-023-00172-3 37275539 PMC10232394

[B41] Hassan M. Abdelnabi H. A. Mohsin S. (2024). Harnessing the potential of PLGA nanoparticles for enhanced bone regeneration. Pharmaceutics 16 (2), 273. 10.3390/pharmaceutics16020273 38399327 PMC10892810

[B42] Hettiarachchi S. D. Zhou Y. Seven E. Lakshmana M. K. Kaushik A. K. Chand H. S. (2019). Nanoparticle-mediated approaches for Alzheimer's disease pathogenesis, diagnosis, and therapeutics. J. Control Release 314, 125–140. 10.1016/j.jconrel.2019.10.034 31647979

[B43] Hossain M. I. Marcus J. M. Lee J. H. Garcia P. L. Singh V. Shacka J. J. (2021). Restoration of CTSD (cathepsin D) and lysosomal function in stroke is neuroprotective. Autophagy 17 (6), 1330–1348. 10.1080/15548627.2020.1761219 32450052 PMC8205033

[B44] Hu H. Liao Z. Xu M. Wan S. Wu Y. Zou W. (2023). Fabrication, optimization, and evaluation of paclitaxel and curcumin coloaded PLGA nanoparticles for improved antitumor activity. ACS Omega 8 (1), 976–986. 10.1021/acsomega.2c06359 36643566 PMC9835547

[B45] Hu Q. Luo Y. (2021). Chitosan-based nanocarriers for encapsulation and delivery of curcumin: a review. Int. J. Biol. Macromol. 179, 125–135. 10.1016/j.ijbiomac.2021.02.216 33667554

[B46] Huse J. T. Liu K. Pijak D. S. Carlin D. Lee V. M. Doms R. W. (2002) Beta-secretase processing in the trans-Golgi network preferentially generates truncated amyloid species that accumulate in Alzheimer's disease brain. J. Biol. Chem. 277 0021–9258. 10.1074/jbc.M11114120 11847218

[B47] Imran H. Tang Y. Wang S. Yan X. Liu C. Guo L. (2023). Optimized dox drug deliveries via chitosan-mediated nanoparticles and stimuli responses in cancer chemotherapy: a review. Molecules 29 (1), 31. 10.3390/molecules29010031 38202616 PMC10780101

[B48] Jack C. R. Jr. Bennett D. A. Blennow K. Carrillo M. C. Dunn B. Haeberlein S. B. (2018). NIA-AA Research Framework: toward a biological definition of Alzheimer's disease. Alzheimers Dement. 14 (4), 535–562. 10.1016/j.jalz.2018.02.018 29653606 PMC5958625

[B49] Jain R. A. (2000). The manufacturing techniques of various drug loaded biodegradable poly(lactide-co-glycolide) (PLGA) devices. Biomaterials 21 (23), 2475–2490. 10.1016/s0142-9612(00)00115-0 11055295

[B50] Jamshidnejad-Tosaramandani T. Kashanian S. Karimi I. Schiöth H. B. (2024). Synthesis of a rivastigmine and insulin combinational mucoadhesive nanoparticle for intranasal delivery. Polym. (Basel) 16 (4), 510. 10.3390/polym16040510 PMC10891873 38399888

[B51] Jeon S. G. Cha M. Y. Kim J. I. Hwang T. W. Kim K. A. Kim T. H. (2019). Vitamin D-binding protein-loaded PLGA nanoparticles suppress Alzheimer's disease-related pathology in 5XFAD mice. Nanomedicine 17, 297–307. 10.1016/j.nano.2019.02.004 30794963

[B52] Ji Y. B. Lee S. Ju H. J. Kim H. E. Noh J. H. Choi S. (2023). Preparation and evaluation of injectable microsphere formulation for longer sustained release of donepezil. J. Control Release 356, 43–58. 10.1016/j.jconrel.2023.02.024 36841288

[B53] Jia S. Lu Z. Gao Z. An J. Wu X. Li X. (2016). Chitosan oligosaccharides alleviate cognitive deficits in an amyloid-β1-42-induced rat model of Alzheimer's disease. Int. J. Biol. Macromol. 83, 416–425. 10.1016/j.ijbiomac.2015.11.011 26601759

[B54] Johnson J. W. Kotermanski S. E. (2006). Mechanism of action of memantine. Curr. Opin. Pharmacol. 6 (1), 61–67. 10.1016/j.coph.2005.09.007 16368266

[B55] Joodi G. Ansari N. Khodagholi F. (2011). Chitooligosaccharide-mediated neuroprotection is associated with modulation of Hsps expression and reduction of MAPK phosphorylation. Int. J. Biol. Macromol. 48 (5), 726–735. 10.1016/j.ijbiomac.2011.02.011 21356235

[B56] Kaehler S. T. Phleps W. Hesse E. (2003). Dexibuprofen: pharmacology, therapeutic uses and safety. Inflammopharmacology 11 (4), 371–383. 10.1163/156856003322699555 15035791

[B57] Kaiser M. Kirsch B. Hauser H. Schneider D. Seuß-Baum I. Goycoolea F. M. (2015). *In vitro* and sensory evaluation of capsaicin-loaded nanoformulations. PLoS One 10 (10), e0141017. 10.1371/journal.pone.0141017 26492045 PMC4619637

[B58] Kakish H. F. Tashtoush B. Ibrahim H. G. Najib N. M. (2002). A novel approach for the preparation of highly loaded polymeric controlled release dosage forms of diltiazem HCl and diclofenac sodium. Eur. J. Pharm. Biopharm. 54 (1), 75–81. 10.1016/s0939-6411(02)00035-8 12084505

[B59] Kaur H. Das T. Kumar R. Ajore R. Bharadwaj L. M. (2008). Covalent attachment of actin filaments to Tween 80 coated polystyrene beads for cargo transportation. Biosystems 92 (1), 69–75. 10.1016/j.biosystems.2007.12.003 18261846

[B60] Kazemi Shariat Panahi H. Dehhaghi M. Amiri H. Guillemin G. J. Gupta V. K. Rajaei A. (2023). Current and emerging applications of saccharide-modified chitosan: a critical review. Biotechnol. Adv. 66, 108172. 10.1016/j.biotechadv.2023.108172 37169103

[B61] Kellar D. Craft S. (2020). Brain insulin resistance in Alzheimer's disease and related disorders: mechanisms and therapeutic approaches. Lancet Neurol. 19 (9), 758–766. 10.1016/s1474-4422(20)30231-3 32730766 PMC9661919

[B62] Kerr J. S. Adriaanse B. A. Greig N. H. Mattson M. P. Cader M. Z. Bohr V. A. (2017). Mitophagy and Alzheimer's disease: cellular and molecular mechanisms. Trends Neurosci. 40 (3), 151–166. 10.1016/j.tins.2017.01.002 28190529 PMC5341618

[B63] Kias F. Bodmeier R. (2024). Acceleration of final residual solvent extraction from poly(lactide-co-glycolide) microparticles. Pharm. Res. 41 (9), 1869–1879. 10.1007/s11095-024-03744-9 39147990 PMC11436459

[B64] Knop K. Hoogenboom R. Fischer D. Schubert U. S. (2010). Poly(ethylene glycol) in drug delivery: pros and cons as well as potential alternatives. Angew. Chem. Int. Ed. Engl. 49 (36), 6288–6308. 10.1002/anie.200902672 20648499

[B65] Lee C. S. Kulkarni Y. Pierre V. Maski M. Wanner C. (2024). Adverse impacts of PEGylated protein therapeutics: a targeted literature review. BioDrugs 38 (6), 795–819. 10.1007/s40259-024-00684-z 39417964 PMC11530478

[B66] Lee D. Minko T. (2021). Nanotherapeutics for nose-to-brain drug delivery: an approach to bypass the blood brain barrier. Pharmaceutics 13 (12), 2049. 10.3390/pharmaceutics13122049 34959331 PMC8704573

[B67] Liu Z. Gao X. Kang T. Jiang M. Miao D. Gu G. (2013). B6 peptide-modified PEG-PLA nanoparticles for enhanced brain delivery of neuroprotective peptide. Bioconjug Chem. 24 (6), 997–1007. 10.1021/bc400055h 23718945

[B68] Lopez-Lee C. Torres E. R. S. Carling G. Gan L. (2024). Mechanisms of sex differences in Alzheimer's disease. Neuron 112 (8), 1208–1221. 10.1016/j.neuron.2024.01.024 38402606 PMC11076015

[B69] Loy C. Schneider L. (2006). Galantamine for Alzheimer's disease and mild cognitive impairment. Cochrane Database Syst. Rev. 2006 (1), Cd001747. 10.1002/14651858.CD001747.pub3 16437436 PMC8961200

[B70] Lu J. Pan Q. Zhou J. Weng Y. Chen K. Shi L. (2022). Pharmacokinetics, distribution, and excretion of sodium oligomannose, a recently approved anti-Alzheimer's disease drug in China. J. Pharm. Anal. 12 (1), 145–155. 10.1016/j.jpha.2021.06.001 35573885 PMC9073255

[B71] Lü J. M. Wang X. Marin-Muller C. Wang H. Lin P. H. Yao Q. (2009). Current advances in research and clinical applications of PLGA-based nanotechnology. Expert Rev. Mol. Diagn 9 (4), 325–341. 10.1586/erm.09.15 19435455 PMC2701163

[B72] Makadia H. K. Siegel S. J. (2011). Poly lactic-co-glycolic acid (PLGA) as biodegradable controlled drug delivery carrier. Polym. (Basel) 3 (3), 1377–1397. 10.3390/polym3031377 PMC3347861 22577513

[B73] Mao Y. F. Guo Z. Zheng T. Jiang Y. Yan Y. Yin X. (2016). Intranasal insulin alleviates cognitive deficits and amyloid pathology in young adult APPswe/PS1dE9 mice. Aging Cell. 15 (5), 893–902. 10.1111/acel.12498 27457264 PMC5013027

[B74] Marques A. R. A. Di Spiezio A. Thießen N. Schmidt L. Grötzinger J. Lüllmann-Rauch R. (2020). Enzyme replacement therapy with recombinant pro-CTSD (cathepsin D) corrects defective proteolysis and autophagy in neuronal ceroid lipofuscinosis. Autophagy 16 (5), 811–825. 10.1080/15548627.2019.1637200 31282275 PMC7158922

[B75] Martin E. Boucher C. Fontaine B. Delarasse C. (2017). Distinct inflammatory phenotypes of microglia and monocyte-derived macrophages in Alzheimer's disease models: effects of aging and amyloid pathology. Aging Cell. 16 (1), 27–38. 10.1111/acel.12522 27723233 PMC5242297

[B76] Mistry A. Glud S. Z. Kjems J. Randel J. Howard K. A. Stolnik S. (2009). Effect of physicochemical properties on intranasal nanoparticle transit into murine olfactory epithelium. J. Drug Target 17 (7), 543–552. 10.1080/10611860903055470 19530905

[B77] Moncalvo F. Martinez Espinoza M. I. Cellesi F. (2020). Nanosized delivery systems for therapeutic proteins: clinically validated technologies and advanced development strategies. Front. Bioeng. Biotechnol. 8, 89. 10.3389/fbioe.2020.00089 32117952 PMC7033645

[B78] Mundargi R. C. Babu V. R. Rangaswamy V. Patel P. Aminabhavi T. M. (2008). Nano/micro technologies for delivering macromolecular therapeutics using poly(D,L-lactide-co-glycolide) and its derivatives. J. Control Release 125 (3), 193–209. 10.1016/j.jconrel.2007.09.013 18083265

[B79] Naveed M. Phil L. Sohail M. Hasnat M. Baig M. Ihsan A. U. (2019). Chitosan oligosaccharide (COS): an overview. Int. J. Biol. Macromol. 129, 827–843. 10.1016/j.ijbiomac.2019.01.192 30708011

[B80] Nojoki F. Ebrahimi-Hosseinzadeh B. Hatamian-Zarmi A. Khodagholi F. Khezri K. (2022). Design and development of chitosan-insulin-transfersomes (Transfersulin) as effective intranasal nanovesicles for the treatment of Alzheimer's disease: *in vitro*, *in vivo*, and *ex vivo* evaluations. Biomed. Pharmacother. 153, 113450. 10.1016/j.biopha.2022.113450 36076565

[B81] Opatha S. A. T. Titapiwatanakun V. Chutoprapat R. (2020). Transfersomes: a promising nanoencapsulation technique for transdermal drug delivery. Pharmaceutics 12 (9), 855. 10.3390/pharmaceutics12090855 32916782 PMC7559928

[B82] Ouyang Q. Q. Zhao S. Li S. D. Song C. (2017). Application of chitosan, chitooligosaccharide, and their derivatives in the treatment of Alzheimer's disease. Mar. Drugs 15 (11), 322. 10.3390/md15110322 29112116 PMC5706020

[B83] Paul P. S. Cho J. Y. Wu Q. Karthivashan G. Grabovac E. Wille H. (2022). Unconjugated PLGA nanoparticles attenuate temperature-dependent β-amyloid aggregation and protect neurons against toxicity: implications for Alzheimer's disease pathology. J. Nanobiotechnology 20 (1), 67. 10.1186/s12951-022-01269-0 35120558 PMC8817552

[B84] Paul P. S. Patel T. Cho J. Y. Yarahmady A. Khalili A. Semenchenko V. (2024). Native PLGA nanoparticles attenuate Aβ-seed induced tau aggregation under *in vitro* conditions: potential implication in Alzheimer's disease pathology. Sci. Rep. 14 (1), 144. 10.1038/s41598-023-50465-x 38167993 PMC10762165

[B85] Porte K. Renoux B. Péraudeau E. Clarhaut J. Eddhif B. Poinot P. (2019). Controlled release of a micelle payload via sequential enzymatic and bioorthogonal reactions in living systems. Angew. Chem. Int. Ed. Engl. 58 (19), 6366–6370. 10.1002/anie.201902137 30856679

[B86] Pottanam Chali S. Ravoo B. J. (2020). Polymer nanocontainers for intracellular delivery. Angew. Chem. Int. Ed. Engl. 59 (8), 2962–2972. 10.1002/anie.201907484 31364243 PMC7028112

[B87] Prvulovic D. Hampel H. Pantel J. (2010). Galantamine for Alzheimer's disease. Expert Opin. Drug Metab. Toxicol. 6 (3), 345–354. 10.1517/17425251003592137 20113148

[B88] Qian Y. Zha Y. Feng B. Pang Z. Zhang B. Sun X. (2013). PEGylated poly(2-(dimethylamino) ethyl methacrylate)/DNA polyplex micelles decorated with phage-displayed TGN peptide for brain-targeted gene delivery. Biomaterials 34 (8), 2117–2129. 10.1016/j.biomaterials.2012.11.050 23245924

[B89] Reisberg B. Doody R. Stöffler A. Schmitt F. Ferris S. Möbius H. J. (2003). Memantine in moderate-to-severe Alzheimer's disease. N. Engl. J. Med. 348 (14), 1333–1341. 10.1056/NEJMoa013128 12672860

[B90] Repnik U. Stoka V. Turk V. Turk B. (2012). Lysosomes and lysosomal cathepsins in cell death. Biochim. Biophys. Acta 1824 (1), 22–33. 10.1016/j.bbapap.2011.08.016 21914490

[B91] Reshma V. G. Syama S. Sruthi S. Reshma S. C. Remya N. S. Mohanan P. V. (2017). Engineered nanoparticles with antimicrobial property. Curr. Drug Metab. 18 (11), 1040–1054. 10.2174/1389200218666170925122201 28952436

[B92] Saito K. Elce J. S. Hamos J. E. Nixon R. A. (1993). Widespread activation of calcium-activated neutral proteinase (calpain) in the brain in Alzheimer disease: a potential molecular basis for neuronal degeneration. Proc. Natl. Acad. Sci. U. S. A. 90 (7), 2628–2632. 10.1073/pnas.90.7.2628 8464868 PMC46148

[B93] Sánchez-López E. Ettcheto M. Egea M. A. Espina M. Calpena A. C. Folch J. (2017). New potential strategies for Alzheimer's disease prevention: pegylated biodegradable dexibuprofen nanospheres administration to APPswe/PS1dE9. Nanomedicine 13 (3), 1171–1182. 10.1016/j.nano.2016.12.003 27986603

[B94] Sánchez-López E. Ettcheto M. Egea M. A. Espina M. Cano A. Calpena A. C. (2018). Memantine loaded PLGA PEGylated nanoparticles for Alzheimer's disease: *in vitro* and *in vivo* characterization. J. Nanobiotechnology 16 (1), 32. 10.1186/s12951-018-0356-z 29587747 PMC5870370

[B95] Saxena M. Dubey R. (2019). Target enzyme in Alzheimer's disease: acetylcholinesterase inhibitors. Curr. Top. Med. Chem. 19 (4), 264–275. 10.2174/1568026619666190128125912 30706815

[B96] Scheltens P. De Strooper B. Kivipelto M. Holstege H. Chételat G. Teunissen C. E. (2021). Alzheimer's disease. Lancet 397 (10284), 1577–1590. 10.1016/s0140-6736(20)32205-4 33667416 PMC8354300

[B97] Schliecker G. Schmidt C. Fuchs S. Wombacher R. Kissel T. (2003). Hydrolytic degradation of poly(lactide-co-glycolide) films: effect of oligomers on degradation rate and crystallinity. Int. J. Pharm. 266 (1-2), 39–49. 10.1016/s0378-5173(03)00379-x 14559392

[B98] Shi L. Zhang J. Zhao M. Tang S. Cheng X. Zhang W. (2021). Effects of polyethylene glycol on the surface of nanoparticles for targeted drug delivery. Nanoscale 13 (24), 10748–10764. 10.1039/d1nr02065j 34132312

[B99] Shi S. Yao C. Cen J. Li L. Liu G. Hu J. (2020). High-fidelity end-functionalization of poly(ethylene glycol) using stable and potent carbamate linkages. Angew. Chem. Int. Ed. Engl. 59 (41), 18172–18178. 10.1002/anie.202006687 32643249

[B100] Shibata M. Yamada S. Kumar S. R. Calero M. Bading J. Frangione B. (2000). Clearance of Alzheimer's amyloid-ss(1-40) peptide from brain by LDL receptor-related protein-1 at the blood-brain barrier. J. Clin. Investig. 106 (12), 1489–1499. 10.1172/jci10498 11120756 PMC387254

[B101] Singer O. Marr R. A. Rockenstein E. Crews L. Coufal N. G. Gage F. H. (2005). Targeting BACE1 with siRNAs ameliorates Alzheimer disease neuropathology in a transgenic model. Nat. Neurosci. 8 (10), 1343–1349. 10.1038/nn1531 16136043

[B102] Smith J. M. Dornish M. Wood E. J. (2005). Involvement of protein kinase C in chitosan glutamate-mediated tight junction disruption. Biomaterials 26 (16), 3269–3276. 10.1016/j.biomaterials.2004.06.020 15603822

[B103] Sonam Dongsar T. Tsering Dongsar T. Gupta G. Alsayari A. Wahab S. Kesharwani P. (2024). PLGA nanomedical consignation: a novel approach for the management of prostate cancer. Int. J. Pharm. 652, 123808. 10.1016/j.ijpharm.2024.123808 38224758

[B104] Stoka V. Turk V. Turk B. (2016). Lysosomal cathepsins and their regulation in aging and neurodegeneration. Ageing Res. Rev. 32, 22–37. 10.1016/j.arr.2016.04.010 27125852

[B105] Suire C. N. Abdul-Hay S. O. Sahara T. Kang D. Brizuela M. K. Saftig P. (2020). Cathepsin D regulates cerebral Aβ42/40 ratios via differential degradation of Aβ42 and Aβ40. Alzheimers Res. Ther. 12 (1), 80. 10.1186/s13195-020-00649-8 32631408 PMC7339583

[B106] Sun R. Chen Y. Pei Y. Wang W. Zhu Z. Zheng Z. (2024). The drug release of PLGA-based nanoparticles and their application in treatment of gastrointestinal cancers. Heliyon 10 (18), e38165. 10.1016/j.heliyon.2024.e38165 39364250 PMC11447355

[B107] Sung H. W. Sonaje K. Liao Z. X. Hsu L. W. Chuang E. Y. (2012). pH-responsive nanoparticles shelled with chitosan for oral delivery of insulin: from mechanism to therapeutic applications. Acc. Chem. Res. 45 (4), 619–629. 10.1021/ar200234q 22236133

[B108] van Steenis J. H. van Maarseveen E. M. Verbaan F. J. Verrijk R. Crommelin D. J. Storm G. (2003). Preparation and characterization of folate-targeted pEG-coated pDMAEMA-based polyplexes. J. Control Release 87 (1-3), 167–176. 10.1016/s0168-3659(02)00361-9 12618033

[B109] Wang F. Gómez-Sintes R. Boya P. (2018b). Lysosomal membrane permeabilization and cell death. Traffic 19 (12), 918–931. 10.1111/tra.12613 30125440

[B110] Wang P. Yang G. Mosier D. R. Chang P. Zaidi T. Gong Y. D. (2005). Defective neuromuscular synapses in mice lacking amyloid precursor protein (APP) and APP-Like protein 2. J. Neurosci. 25 (5), 1219–1225. 10.1523/jneurosci.4660-04.2005 15689559 PMC6725967

[B111] Wang P. Zheng X. Guo Q. Yang P. Pang X. Qian K. (2018a). Systemic delivery of BACE1 siRNA through neuron-targeted nanocomplexes for treatment of Alzheimer's disease. J. Control Release 279, 220–233. 10.1016/j.jconrel.2018.04.034 29679667

[B112] Wang T. Kuang W. Chen W. Xu W. Zhang L. Li Y. (2020b). A phase II randomized trial of sodium oligomannate in Alzheimer's dementia. Alzheimers Res. Ther. 12 (1), 110. 10.1186/s13195-020-00678-3 32928279 PMC7489025

[B113] Wang X. Sun G. Feng T. Zhang J. Huang X. Wang T. (2019). Sodium oligomannate therapeutically remodels gut microbiota and suppresses gut bacterial amino acids-shaped neuroinflammation to inhibit Alzheimer's disease progression. Cell. Res. 29 (10), 787–803. 10.1038/s41422-019-0216-x 31488882 PMC6796854

[B114] Wang X. Xie Z. Yuan J. Jin E. Lian W. Chang S. (2024). Sodium oligomannate disrupts the adherence of Rib(high) bacteria to gut epithelia to block SAA-triggered Th1 inflammation in 5XFAD transgenic mice. Cell. Discov. 10 (1), 115. 10.1038/s41421-024-00725-5 39557828 PMC11573985

[B115] Wang Y. Wu Q. Anand B. G. Karthivashan G. Phukan G. Yang J. (2020a). Significance of cytosolic cathepsin D in Alzheimer's disease pathology: protective cellular effects of PLGA nanoparticles against β-amyloid-toxicity. Neuropathol. Appl. Neurobiol. 46 (7), 686–706. 10.1111/nan.12647 32716575 PMC8202514

[B116] Wolfe D. M. Lee J. H. Kumar A. Lee S. Orenstein S. J. Nixon R. A. (2013). Autophagy failure in Alzheimer's disease and the role of defective lysosomal acidification. Eur. J. Neurosci. 37 (12), 1949–1961. 10.1111/ejn.12169 23773064 PMC3694736

[B117] Won Y. Y. Sharma R. Konieczny S. F. (2009). Missing pieces in understanding the intracellular trafficking of polycation/DNA complexes. J. Control Release 139 (2), 88–93. 10.1016/j.jconrel.2009.06.031 19580830 PMC3336099

[B118] Wu J. Wang Y. Yang H. Liu X. Lu Z. (2017). Preparation and biological activity studies of resveratrol loaded ionically cross-linked chitosan-TPP nanoparticles. Carbohydr. Polym. 175, 170–177. 10.1016/j.carbpol.2017.07.058 28917853

[B119] Wu Q. Karthivashan G. Nakhaei-Nejad M. Anand B. G. Giuliani F. Kar S. (2022). Native PLGA nanoparticles regulate APP metabolism and protect neurons against β-amyloid toxicity: potential significance in Alzheimer's disease pathology. Int. J. Biol. Macromol. 219, 1180–1196. 10.1016/j.ijbiomac.2022.08.148 36030976

[B120] Xiao S. Chan P. Wang T. Hong Z. Wang S. Kuang W. (2021). A 36-week multicenter, randomized, double-blind, placebo-controlled, parallel-group, phase 3 clinical trial of sodium oligomannate for mild-to-moderate Alzheimer's dementia. Alzheimers Res. Ther. 13 (1), 62. 10.1186/s13195-021-00795-7 33731209 PMC7967962

[B121] Xu J. Patassini S. Begley P. Church S. Waldvogel H. J. Faull R. L. M. (2020). Cerebral deficiency of vitamin B5 (d-pantothenic acid; pantothenate) as a potentially-reversible cause of neurodegeneration and dementia in sporadic Alzheimer's disease. Biochem. Biophys. Res. Commun. 527 (3), 676–681. 10.1016/j.bbrc.2020.05.015 32416962

[B122] Yang L. Wang Y. Li Z. Wu X. Mei J. Zheng G. (2023). Brain targeted peptide-functionalized chitosan nanoparticles for resveratrol delivery: impact on insulin resistance and gut microbiota in obesity-related Alzheimer's disease. Carbohydr. Polym. 310, 120714. 10.1016/j.carbpol.2023.120714 36925241

[B123] Yeo-Teh N. S. L. Tang B. L. (2023). A review of scientific ethics issues associated with the recently approved drugs for Alzheimer's disease. Sci. Eng. Ethics 29 (1), 2. 10.1007/s11948-022-00422-0 36625928

[B124] Younes I. Rinaudo M. (2015). Chitin and chitosan preparation from marine sources. Structure, properties and applications. Mar. Drugs 13 (3), 1133–1174. 10.3390/md13031133 25738328 PMC4377977

[B125] Zatsepin T. S. Kotelevtsev Y. V. Koteliansky V. (2016). Lipid nanoparticles for targeted siRNA delivery - going from bench to bedside. Int. J. Nanomedicine 11, 3077–3086. 10.2147/ijn.S106625 27462152 PMC4939975

[B126] Zhang C. Wan X. Zheng X. Shao X. Liu Q. Zhang Q. (2014). Dual-functional nanoparticles targeting amyloid plaques in the brains of Alzheimer's disease mice. Biomaterials 35 (1), 456–465. 10.1016/j.biomaterials.2013.09.063 24099709

[B127] Zhang L. Zhang Z. Li C. Hu Z. Liang Y. Yang Z. (2022). Preparation and characterization of amphiphilic chitosan/iodine composite film as antimicrobial material. Int. J. Biol. Macromol. 222 (Pt B), 2426–2438. 10.1016/j.ijbiomac.2022.10.028 36220406

[B128] Zhang Y. Chan H. F. Leong K. W. (2013). Advanced materials and processing for drug delivery: the past and the future. Adv. Drug Deliv. Rev. 65 (1), 104–120. 10.1016/j.addr.2012.10.003 23088863 PMC3565095

[B129] Zheng K. Dai X. Xiao N. Wu X. Wei Z. Fang W. (2017a). Curcumin ameliorates memory decline via inhibiting BACE1 expression and β-amyloid pathology in 5×FAD transgenic mice. Mol. Neurobiol. 54 (3), 1967–1977. 10.1007/s12035-016-9802-9 26910813

[B130] Zheng X. Pang X. Yang P. Wan X. Wei Y. Guo Q. (2017b). A hybrid siRNA delivery complex for enhanced brain penetration and precise amyloid plaque targeting in Alzheimer's disease mice. Acta Biomater. 49, 388–401. 10.1016/j.actbio.2016.11.029 27845275

[B131] Zhou Y. Y. Du Y. Z. Wang L. Yuan H. Zhou J. P. Hu F. Q. (2010). Preparation and pharmacodynamics of stearic acid and poly (lactic-co-glycolic acid) grafted chitosan oligosaccharide micelles for 10-hydroxycamptothecin. Int. J. Pharm. 393 (1-2), 143–151. 10.1016/j.ijpharm.2010.04.025 20420886

